# Traction-assisted and saline immersion endoscopic submucosal dissection for complete resection of peri-appendiceal large nonpedunculated colorectal polyps

**DOI:** 10.1055/a-2533-2996

**Published:** 2025-02-20

**Authors:** Michele Montori, Maria E. Argenziano, Pieter J. Poortmans, Sander Smeets, Andrea Sorge, Luca Maroni, David J. Tate

**Affiliations:** 1 Clinic of Gastroenterology, Hepatology and Emergency Digestive Endoscopy, Università Politecnica delle Marche, Ancona, Italy; 2 60200 Department of Gastroenterology and Hepatology, University Hospital Ghent, Gent, Belgium; 3 Faculty of Medicine and Health Sciences, University of Ghent, Gent, Belgium; 4 Department of Gastroenterology and Hepatology, University Hospital Brussels, Brussel, Belgium; 5 9304 Department of Pathophysiology and Transplantation, University of Milan, Milan, Italy; 6 9294 Clinic of Gastroenterology, Hepatology and Emergency Digestive Endoscopy, Università Politecnica delle Marche, Ancona, Italy

Endoscopic resection can be challenging for large nonpedunculated colorectal polyps (LNPCPs) around the appendiceal orifice. Factors predicting failure of endoscopic mucosal resection (EMR) include deep invasion of the appendiceal orifice and >50% involvement of the appendiceal orifice. In contrast, patients with a previous appendectomy are often able to undergo complete resection using EMR for LNPCPs that completely cover the prior appendiceal orifice 1 . Unfortunately, it is often difficult to tell upfront which of these factors is present in a given LNPCP.

Other options include full-thickness resection, which carries a 25% risk of acute appendicitis and is not suitable for large lesions 2 . Surgery may also be considered, but an extended appendectomy is often insufficient for lesions extending into the cecum. More extensive surgery, such as ileocecal resection or right hemicolectomy, carry significant risks, including 20% morbidity and 0.5% mortality 3 .

This case series ( Video 1 ) demonstrates endoscopic submucosal dissection (ESD) for peri-appendiceal LNPCPs showing type 3a, 1, and 3 lesions according to Toyonaga’s classification 3 .


Multipoint traction is applied to lift and expose the appendix, facilitating clear visualization and access to the dissection plane. Source for graphical illustration: Created in BioRender. Vanhooren, M. (2025) https://BioRender.com/a33w603 [rerif].
Video 1

Other studies have described the ESD technique for LNPCPs of the appendiceal orifice using both traction-assisted and nontraction methods 3 4 5 . However, none have described a combined approach using multipoint traction with saline immersion.

In this case series, multipoint traction, using a multiband system ( Fig. 1 ), is applied to lift the appendix and expose the submucosal plane between the appendiceal mucosa and muscularis propria ( Video 1 ) for precise dissection. Saline immersion is used intermittently to improve visualization and access to the dissection plane ( Fig. 2 ).

**Fig. 1:**
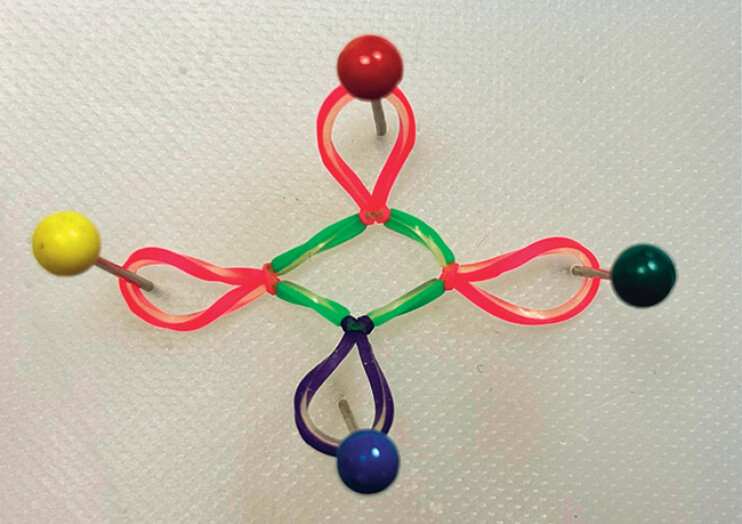
The multiband system is applied with pink bands attached to the lesion margin and purple bands secured to an opposite fold, with the green band serving as a connection between the bands.

**Fig. 2:**
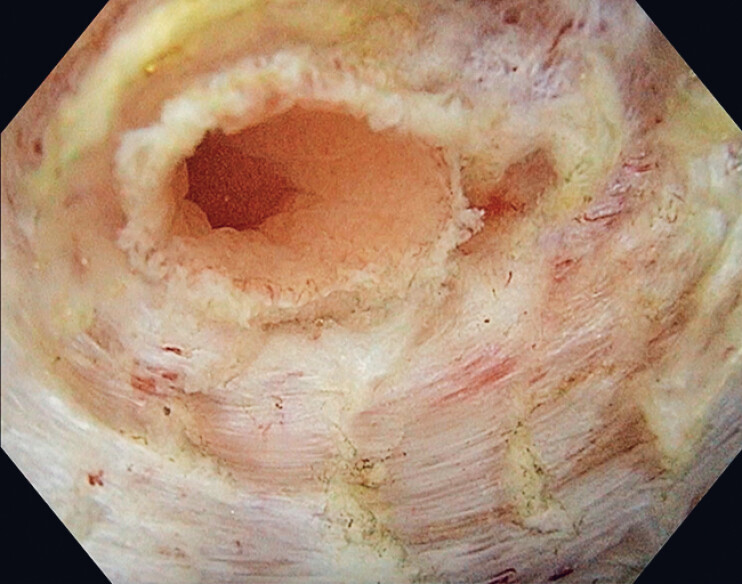
Assessment of the defect margins is performed in saline immersion and with magnification to ensure clear visualization and precise evaluation of the resection area.

In contrast to EMR, even lesions with deep invasion into the appendiceal orifice can be fully resected using this ESD-based technique as the caudal appendiceal margin can be visualized and the depth of mucosal incision within the appendix completely controlled. Furthermore, lesions fully encircling the orifice can also be completely removed.

In conclusion, using traction-assisted ESD with intermittent saline immersion, the skilled practitioner can avoid the pitfalls of EMR while avoiding the excess risks of full-thickness resection and laparoscopic surgery.

Endoscopy_UCTN_Code_TTT_1AQ_2AD_3AD

## Notes

Endoscopy E-Videos https://eref.thieme.de/e-videos
*E-Videos* is an open access online section of the journal *Endoscopy* , reporting on interesting cases and new techniques in gastroenterological endoscopy. All papers include a high-quality video and are published with a Creative Commons CC-BY license. Endoscopy E-Videos qualify for HINARI discounts and waivers and eligibility is automatically checked during the submission process. We grant 100% waivers to articles whose corresponding authors are based in Group A countries and 50% waivers to those who are based in Group B countries as classified by Research4Life (see: https://www.research4life.org/access/eligibility/ ). This section has its own submission website at https://mc.manuscriptcentral.com/e-videos .
